# Effects of Mangrove (*Avicennia marina*) Leaf Aqueous Extract on Growth, Immunity, and Hypoxia Tolerance in Gray Mullet (*Liza ramada*)

**DOI:** 10.1155/anu/2381301

**Published:** 2025-05-24

**Authors:** Mohammed F. El Basuini, Esraa Roshdi, Tamer El-Sayed Ali, Salma M. Zeid, Ali A. Soliman, Mahmoud S. Gewaily, Islam I. Teiba, Mayada Alhoshy, Emad H. El-Bilawy, Islam Mamdouh, Akram Ismael Shehata

**Affiliations:** ^1^Faculty of Agriculture, Tanta University, Tanta 31527, Egypt; ^2^King Salman International University, South Sinai 46618, Egypt; ^3^Oceanography Department, Faculty of Science, Alexandria University, Alexandria, Egypt; ^4^Fish Nutrition Laboratory, Aquaculture Division, National Institute of Oceanography and Fisheries, Alexandria 21556, Egypt; ^5^Department of Anatomy and Embryology, Faculty of Veterinary Medicine, Kafrelsheikh University, Kafr El-Sheikh 33516, Egypt; ^6^Independent Researcher, Alexandria, Egypt; ^7^Department of Animal and Fish Production, Faculty of Agriculture (Saba Basha), Alexandria University, Alexandria 21531, Egypt

**Keywords:** antioxidant enzymes, *Avicennia marina*, digestive health, hypoxia tolerance, immune function, *Liza ramada*

## Abstract

This study evaluates the influences of dietary mangrove (*Avicennia marina*) leaf aqueous extract (MLAE) on growth, health, and stress tolerance in *Liza ramada* over an 84-day feeding assessment. Fish (initial weight: 34.89 ± 0.15 g) were served diets encompassing 0, 100, 200, 300, or 400 mg/kg MLAE. The 300 mg/kg MLAE group reached the best growth performance compared to other groups (*p* < 0.05). The feed conversion ratio (FCR) was also the most productive at this level. Polynomial regression identified an optimal MLAE range of 250–275 mg/kg for maximizing growth and feed efficiency. Digestive enzyme potencies (amylase, lipase, and protease) were elevated (*p* < 0.05) in the 300 and 400 mg/kg groups. Higher MLAE levels reduced total bacterial count and populations of *Vibrio* spp. and *Escherichia coli*. Histological analysis showed enhanced intestinal villi structure and immune cell infiltration in MLAE-fed groups. Blood chemistry revealed increased total protein (TP), albumin (AB), and globulin contents and reduced cholesterol in the 200–400 mg/kg groups. MLAE improved hypoxia tolerance, indicated by increased LT50% values, and reduced stress markers (glucose, cortisol) under hypoxia. Immune indicators (lysozyme activity, bactericidal activity [BA], and NBT%) and antioxidant enzyme activities (superoxide dismutase [SOD], catalase [CAT], glutathione peroxidase [GPx]) were enhanced, with lower malondialdehyde (MDA) levels. These outcomes suggest that MLAE supplementation enhances growth, health, and stress resilience in gray mullets, offering potential applications for sustainable aquaculture. Further studies should explore the mechanistic pathways underlying these benefits and assess the long-term impacts of MLAE supplementation on fish health and aquaculture productivity.

## 1. Introduction

Aquaculture has undergone remarkable expansion in recent decades to meet the growing global demand for fish protein [1]. However, this intensification has introduced challenges, including stress-induced physiological disorders, compromised immune functions, and increased susceptibility to diseases in cultured fish species [2]. Among these stressors, hypoxia, a condition of reduced oxygen availability, poses a significant threat to aquaculture sustainability due to its adverse results on fish growth, metabolism, immune responses, and overall health [3, 4]. To address these challenges, strategies that mitigate hypoxia-induced stress and enhance fish resilience have become a priority in aquaculture research [5].

One promising approach is dietary supplementation with natural plant-based bioactive compounds, known for their multifunctional properties, including antioxidant, immunostimulatory, and stress-mitigating effects [6, 7]. Unlike synthetic chemicals or antibiotics, plant-based additives are natural, biodegradable, and environmentally friendly, aligning with the principles of sustainable aquaculture [8]. Additionally, they may help reduce selective pressure for antibiotic resistance, a growing global concern in aquaculture and human health [9]. Plant extracts exhibit diverse bioactivities, including antimicrobial, antioxidant, and immunomodulatory effects, which are critical for improving the health and productivity of cultured fish species [10, 11].

Mangrove plants, particularly *Avicennia marina*, have long been valued in traditional medicine for their therapeutic properties. Extracts from mangroves demonstrate significant therapeutic activities, covering anti-inflammatory, antimicrobial, antioxidant, and immunomodulatory outcomes, ascribed to bioactive constituents comprising flavonoids, polyphenols, saponins, and tannins [12–14]. Mangrove (*A. marina*) leaf aqueous extract (MLAE) has shown potential for enhancing resilience against oxidative stress and infections, making them promising candidates for aquafeed formulations [15]. Although mangrove-derived compounds have demonstrated extensive pharmacological potential in terrestrial applications, their use in aquaculture remains largely unexplored, particularly for economically significant species such as gray mullet (*Liza ramada*).

*L. ramada* (gray mullet) is widely farmed in coastal aquaculture systems due to its adaptability, high nutritional value, and economic significance [16]. However, like many other cultured fish, it is susceptible to hypoxia stress, which can impair growth status, nutrient utilization, digestive function, and immune responses while disrupting gut microbiota and histological integrity of the liver and gastrointestinal tract [17]. Recent investigation suggests that plant-based additives can mitigate these effects by modulating key physiological and biochemical parameters, including antioxidant enzyme activity, blood chemistry, and stress markers [18]. Additionally, plant-derived bioactive compounds have been shown to enhance feed utilization and digestive enzyme potency, contributing to improved growth and health outcomes [19].

This trial evaluates the impacts of dietary MLAE on growth, feed utilization, digestive enzyme, gut microbiota composition, liver and gastrointestinal tract histology, blood chemistry, antioxidant activity, and immunity in gray mullet (*L. ramada*). Furthermore, it investigates the ability of MLAE to mitigate hypoxia-induced stress by modulating stress biomarkers.

## 2. Materials and Methods

### 2.1. Processing and Composition of MLAE

**Figure 1:**
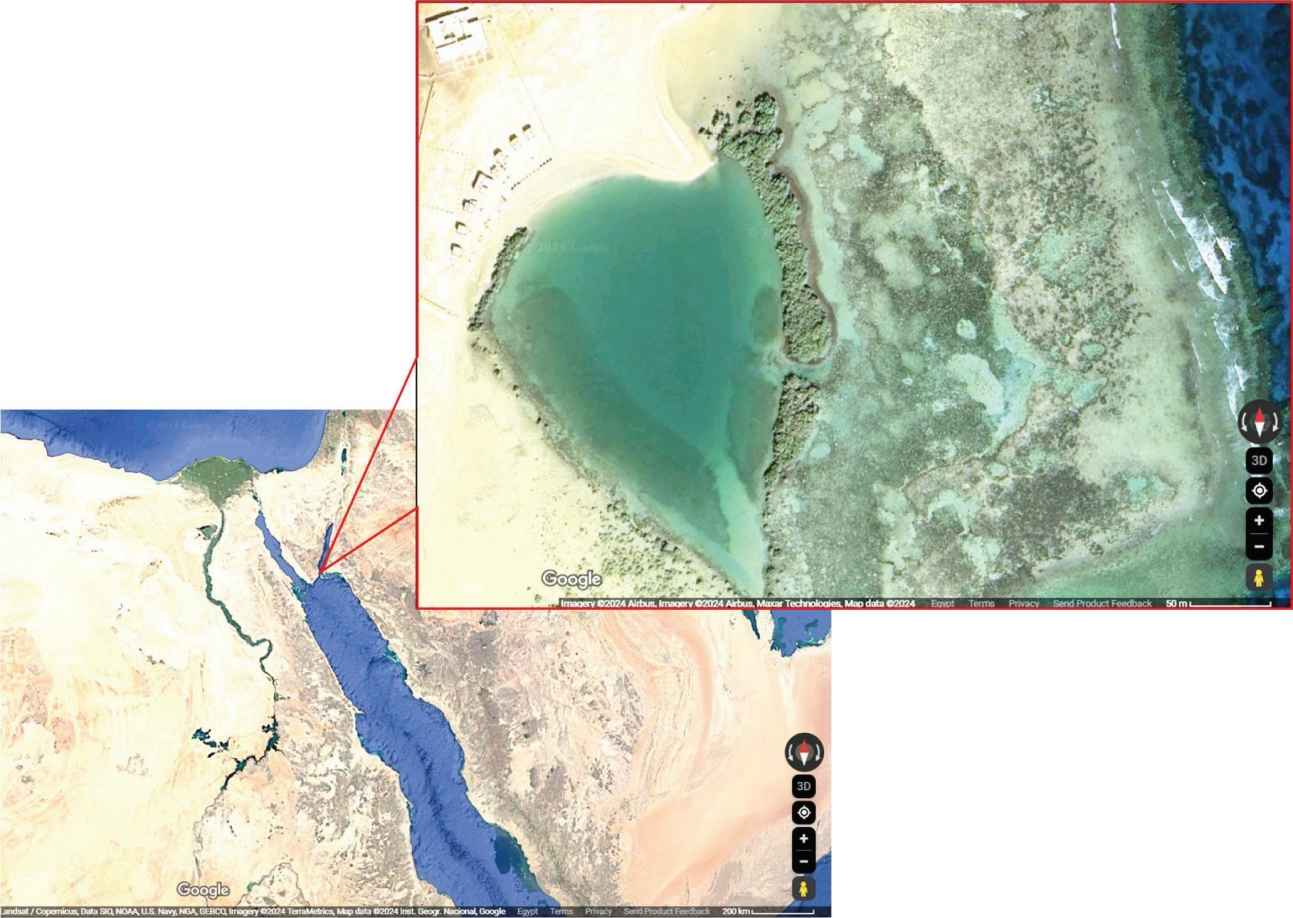
Mangrove (*Avicennia marina*) sampling station map. Nabq Nature Reserve, Egypt, at geographical coordinates (28.12093°N, 34.44183°E).

Mangrove leaves (*A. marina*) were collected from the Nabq Nature Reserve, Egypt, located at geographical coordinates “28.12093°N, 34.44183°E” (Figure 1). The leaves were air-dried and then finely ground with a mortar and pestle. The aqueous extract was prepared following the infusion method described by Fernandez [20]. During this procedure, the ground leaves were soaked in filtered water at a 1:2 (*w*/*v*) for 24 h. The blend was then passed through the Whatman No. 1 filter with the aid of a suction device assembled using a rotary evaporator at 40°C. The concentrated extract was kept in the sterile tube at 4°C and utilized within 1 week to maintain its stability.

The chemical constituents of MLAE were evaluated using a Trace GC Ultra/ISQ Single Quadrupole Mass Spectrometry system (Thermo Scientific, USA) fitted with a TG-5 MS fused silica capillary column (30 m × 0.25 mm × 0.1 µm film thickness). The temperature of the column oven was primarily set at 150°C for 4 min, then heightened to 280°C at a rate of 5°C/min, and held at 280°C for another 4 min. Both the injector and the MS transfer line were maintained at 280°C. Helium served as the carrier gas at a constant flow rate of 1 mL/min. Mass spectra were obtained using electron ionization at 70 eV. The chemical constituents of MLAE were detected by relating their retention times (RTs) and mass spectra with reference data from the NIST and WILEY spectral libraries, following the method described by Abd El-Kareem et al. [21].

### 2.2. Experimental Design

Gray mullets (*L. ramada*) in good health were sourced from a private producer in Kafr Elsheikh governorate and transported to Baltim research unite, NIOF, Egypt, where the feeding phase was conducted. Upon arrival, the fish were adapted for 14 days in a concrete container (5 × 10 × 1 m) supported with constant aeration. Following this phase, the fish were randomly assigned to 15 tanks (2 × 5 × 1 m), each housing 45 fish (initial weight: 34.89 ± 0.15 g).

The feeding stage was conducted over 84 days under a 12-h light/dark sequence with constant ventilation. Throughout the experiment, key water quality parameters were regularly monitored and sustained: temperature (25.34 ± 0.22°C), dissolved oxygen (6.71 ± 0.31 mg/L), pH (7.62 ± 0.54), and total ammonia (0.02 ± 0.001 mg/L). Measurements were taken daily using portable probes and spectrophotometric techniques. The fish were hand-fed to satiation at 07:00 a.m., 01:00 p.m., and 07:00 p.m.

The experimental diets consisted of a control (0 mg/kg MLAE) and four MLAE-supplemented diets (100, 200, 300, and 400 mg MLAE/kg). All diets contained 32.23% ± 0.17% crude protein and 7.19% ± 0.19% crude fat (Table 1), formulated based on previous recommendations [16]. The ingredients were thoroughly blended, formed into pellets (3 mm), air-dehydrated, and kept at 4°C. The proximate composition was determined following AOAC [22] protocols: moisture content by oven dehydrating at 105°C, ash by combustion at 550°C, lipids via Soxhlet technique, protein using the Kjeldahl procedure, and fiber according to the procedure described by Van Soest et al. [23]. Fish care and handling complied with the ethical standards for experimental animal research in accordance with the ARRIVE guidelines version 2.0 and approved by the ethical approval committee of Desert Agriculture, KSIU, Egypt, under reference number (KSIU/2025/DA-3).

### 2.3. Sampling

At the conclusion of the feeding stage, fish were fasted for 24 h, and nine fish from each tank were unbiasedly chosen for sampling. Prior to sampling, the fish were sedated with 100 mg/L of MS-222 (Sigma–Aldrich Co. LLC.). Blood was collected from the caudal vessels using nonheparinized syringes, allowed to clot for ~30 min, and subsequently centrifuged at 3500 *g* for 5 min at 4°C to separate the serum. The serum was immediately snap-frozen in liquid nitrogen and then stored at −80°C for subsequent biochemical and enzyme analyses. The digestive tracts were dissected, cleaned with phosphate-buffered saline (pH 7.5; 1 g per 10 mL) to remove any residues, and homogenized at 4°C. The homogenates were centrifuged at 5000 *g* for 5 min, and the supernatant was collected and stored at 4°C for digestive enzyme activity assays. Additionally, the intestines and livers were excised, fixed in 10% formalin, and preserved for histological analysis.

### 2.4. Performance Indices

The initial (*W*_0 day_) and final body weight (*W*_84 day_) of the fish, total length (TL), and the viscera weight (VW), liver weight (LW), and intestines weight (IW) were recorded to calculate the following indices:

  $$\begin{array}{c} \text{Weight gain}\left(\text{WG},g/\text{fish}\right)=W_{84\text{ day}}-W_{0}\text{ day}, \end{array}$$

  $$\begin{array}{c} \text{Specific growth rate }\left(\text{SGR},\%/\text{day}\right)=\frac{\text{Ln }W_{84\text{ day}}-\text{Ln }W_{\text{0 day}}}{\text{Time},\text{ day}}\times 100, \end{array}$$

  $$\begin{array}{c} \text{Survival rate }\left(\text{SR},\%\right)=\frac{\text{Final fish count}}{\text{Initial fish count}}\times 100, \end{array}$$

  $$\begin{array}{c} \text{Fulton's condition factor }\left(K\text{ factor}\right)=\frac{\text{Body weight}}{{\text{Length}}^{3}}\times 100, \end{array}$$

  $$\begin{array}{c} \text{Feed intake }\left(\text{FI},g/\text{ fish}/t,\text{ day}\right)=\frac{\text{Feed served }-\text{uneaten feed}}{\text{Fish count}}. \end{array}$$

Uneaten feed was recovered using siphoning to minimize variability in feed intake (FI) measurement.

  $$\begin{array}{c} \text{Feed conversion ratio }\left(\text{FCR}\right)=\frac{\text{FI},g}{\text{WG},g}, \end{array}$$

  $$\begin{array}{c} \text{Hepatosomatic index }\left(\text{HSI},\%\right)=\frac{\text{LV},g}{W_{84\text{ day}},g}\times 100, \end{array}$$

  $$\begin{array}{c} \text{Viscerosomatic index }\left(\text{VSI},\%\right)=\frac{\text{VW},g}{W_{84\text{ day}},g}\times 100, \end{array}$$

  $$\begin{array}{c} \text{Intestinosomatic index }\left(\text{ISI},\%\right)=\frac{\text{IW},g}{W_{84\text{ day}},g}\times 100. \end{array}$$

### 2.5. Digestive Enzyme and Microbiota

Digestive enzyme potency and microbial composition were evaluated using established protocols. Protease efficacy was determined using Sigma's assay kit with casein as the reactant [24], while lipase and amylase potentials were measured spectrophotometrically at 540 and 714 nm, respectively [25]. For microbial analysis, intestines from five fish per tank were rinsed, homogenized, and serially diluted in peptone saline (10⁻³ to 10⁻^7^). Total bacterial counts were measured on plate count agar, *Escherichia coli* on fecal coliform agar (ISO 9308-1:1990), *Vibrio* spp. on thiosulfate citrate bile salts sucrose (TCBS) agar [26], and lactic acid bacteria on MRS medium [27]. Bacterial colonies were initially detected based on morphological features (e.g., colony shape, color, and Gram staining), followed by biochemical identification using tests such as catalase (CAT), oxidase, and H_2_S production [28].

### 2.6. Histological Assessment

A histological examination was done immediately after the trial concluded. The collected tissues (liver and intestinal) were fixed in 10% buffered formalin for 48 h. After fixation, the samples underwent dehydration through sorted ethanol sequences, cleared with xylene, and fixed in paraffin wax. Tissue sections measuring 5 µm in thickness were sliced using a rotary microtome (RM2035; Leica Microsystems, Wetzlar, Germany). The sections were then stained with hematoxylin and eosin (H&E) to examine the general tissue morphology and cellular structure [29]. Images of the stained slides were captured with an EC3 camera (Leica, Germany) attached to the Leica DM500 microscope.

### 2.7. Blood Chemistry

Serum biochemical parameters were examined using Bio-Diagnostic kits (Cairo, Egypt) according to the producer's instructions, with catalog numbers as follows: total protein (TP 20 20), albumin (AB 10 10), globulin (calculated by subtracting AB from TP), total cholesterol (TC 20 10), triglycerides (TGs 20 11), alanine aminotransferase (ALT) (AT 10 34), aspartate aminotransferase (AST) (AT 10 45), urea (UR 21 10), and creatinine (CR 12 50). Each test was performed following standardized and enzymatic methods to ensure accurate and reliable results.

### 2.8. Immunity and Antioxidants

Lysozyme efficiency was determined using a turbidometric assay that measures changes in the turbidity of *Micrococcus lysodeikticus* suspensions, as described by Demers and Bayne [30]. The bactericidal activity (BA) was evaluated by measuring growth inhibition of *E. coli* by serum samples after 24-h incubation at 37°C using spectrophotometric analysis at 570 nm wavelength [31]. The inhibition rate was calculated as follows:

$\text{BA }\left(\%\right)=\frac{\text{OD control }-\text{OD sample}}{\text{OD control}}\times 100$ ,

where OD represents optical density.

The neutrophil oxidative burst was quantified using the NBT reduction procedure [32].

Serum antioxidants levels were evaluated using Nanjing Jiancheng diagnostic kits (Bioengineering, China) according to the manufacturer's protocol, with catalog numbers as follows: superoxide dismutase (SOD) (A001-3-2) using WST-1 reagent at 550 nm, CAT (A007-1-1) with ammonium molybdate reagent at 280 nm, glutathione peroxidase (GPx) (A005-1-2) at 412 nm, and malondialdehyde (MDA) (A003-1-1) using thiobarbituric acid reagent at 532 nm.

### 2.9. Hypoxia Challenge and Stress Markers

The hypoxia stress was conducted at the conclusion of the trial, following the procedure outlined by El Basuini et al. [31]. Twenty fish per tank were stocked into 100-L fiberglass tanks without aeration while gradually lowering the tank's water volume until the dorsal fins of the fish became visible above the water surface [33]. Mortality was documented in each tank at regular 10-min intervals until 50% death (death of 10 fish) was reached. The lethal time to 50% mortality (LT50) was calculated in accordance with Moe et al. [34]. During the trial, water quality parameters were recorded as follows: temperature, 25.34 ± 0.21°C; dissolved oxygen, 1.17 ± 0.03 mg/L; pH, 6.52 ± 0.18; and total ammonia, 0.23 ± 0.01 mg/L. Prior to the hypoxia test, nine fish per tank (not subjected to hypoxia) were randomly sampled for baseline blood collection. Following the hypoxia challenge, six surviving fish per tank were sampled for blood collection. Glucose and cortisol levels were measured to assess stress responses before and after the hypoxia challenge, using the EMEG Automatic Analyzer Model 2000 Evolution and Bayer Diagnostics Reagent strips (Spinreact Co., Spain), following the manufacturer's instructions.

### 2.10. Statistical Evaluation

Data was stated as mean ± standard error (SE) from triplicate measurements. Statistical comparisons among treatment groups were achieved using one-way ANOVA with a significance level set at *p* < 0.05, following tests for normality (Shapiro–Wilk) and homogeneity of variance (Levene's test). Duncan's multiple range test was applied for post hoc comparisons to identify significant differences between group means. Polynomial regression test determined the optimal MLAE supplementation dose based on specific growth rate (SGR) and feed conversion ratio (FCR). Paired *t*-tests were used to analyze pre- and posthypoxia glucose and cortisol levels. All statistical computations were accomplished using SPSS (version 20, IBM, USA).

## 3. Results

### 3.1. Chemical Profiling of Mangrove Leaf Extract

**Figure 2:**
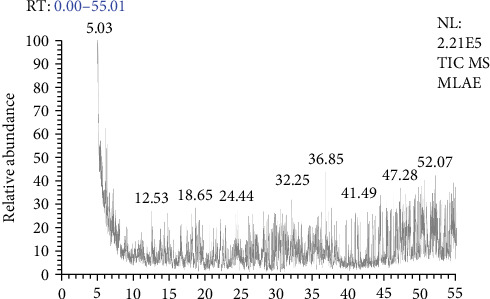
GC–MS chromatogram of mangrove leaf (*Avicennia marina*) aqueous extract.

The GC–MS analysis of the MLAE identified a total of 43 compounds, each characterized by distinct RTs, molecular formulas, masses (*m*/*z*), and areas (%) (Figure 2 and Table 2). These compounds span multiple chemical categories, including esters, phenols, porphyrins, organometallic complexes, aromatic hydrocarbons, halogenated compounds, peptides, and various organic molecules. This diversity highlights the rich chemical composition of MLAE and its potential for diverse biological activities.

Among the identified compounds, porphyrin derivatives were the most prevalent category. Notable examples include (2,3 Dihydro 2 nitro 5,10,15,20 tetraphenyl [3 (2)H1] prophyrinato) copper (II) and Dimethyl 3,3′ [7” (dimethylcarbamoylmethyl) 8″ [4″ hydroxybut 1″ yl) 2″,7″,12″,18″ tetramethyl 7″,8″ dihydro 21H.23H porphyrin 13”,17” diyl] dipropionate, which may contribute to light absorption, enzymatic functions, and other bioactivities. Additionally, organometallic complexes, such as [(Cyclopentadienyl) tris (diethylphosphito P) cobalt O, O“, O”] trichlorozirconium, were also identified. Another prominent group is the halogenated compounds, including 2,5 Dibromo 1,4 di n hexadecylbenzene and 4,4′, 4”,4^′”^ Tetrabromotetraphenylmethane. Furthermore, phenolic compounds, such as o-Cresol (2-methylphenol), and flavonoid derivatives, such as Tetra-O-Methyl-3-O-acetylrobinetinidol, were also identified. Additionally, peptides like YGRKKRRQRRRGPVKRRLFG were detected. Other notable findings include brominated and chlorinated organic molecules, such as N, N′-Bis[3-methoxy-4-hydroxy-5-bromobenzylidene (cyano)acetyl]-1,4-butanediamine.

### 3.2. Performance Indices

The growth indicators, feed efficacy, survival %, and biometric indices of *L. ramada*-fed diets augmented with MLAE at 0, 100, 200, 300, and 400 mg/kg for 84 days are shown in Table 3. Fish-fed diets supplemented with 300 mg/kg MLAE demonstrated the highest growth, with greater *W*_84 day_, WG %, and SGR compared to all other groups. Fish-fed diets enriched with 200 and 400 mg/kg also showed improved growth parameters, though to a lesser extent, while the 100 mg/kg group exhibited comparable performance to the control. FI was significantly higher in fish fed 300 mg/kg MLAE, followed by the 200 and 400 mg/kg groups, all of which consumed more feed than the control and 100 mg/kg groups (*p* < 0.05). FCRs were most efficient in fish fed 300 mg/kg MLAE, followed by those fed 200 mg/kg. The FCR in these groups was significantly lower than that of the control group. Survival rates were high across all groups and showed no differences (*p* > 0.05). Biometric markers, including HSI, ISI, VSI, and *K*-factor, remained unaffected by MLAE supplementation at all levels.

**Figure 3:**
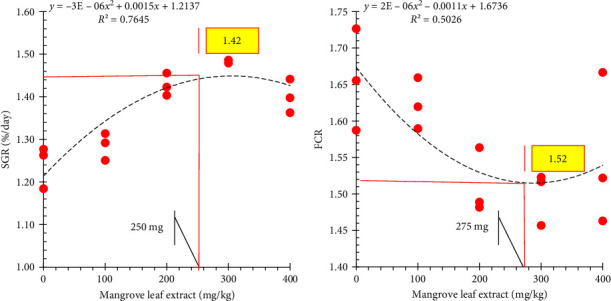
Polynomial regression analyses of specific growth rate (SGR) and feed conversion ratio (FCR) on mangrove leaf (*Avicennia marina*) aqueous extract.

The polynomial regression assessment for SGR and FCR displays the ideal range of mangrove leaf extract supplementation (250–275 mg/kg) that maximizes the growth performance and feed efficiency of the gray mullet (Figure 3).

### 3.3. Digestive Enzyme and Microbiota

The gastrointestinal enzyme capacity of *L. ramada* after an 84-day feeding time with diets augmented with MLAE is presented in Table 4. Amylase activity was enhanced (*p* < 0.05) in fish-fed diets containing MLAE at 300 and 400 mg/kg, with the best activity observed at 400 mg MLAE/kg. The 100 and 200 mg/kg groups exhibited amylase activity comparable to the control. Lipase activity showed a similar pattern, with significant increases observed in the 300 and 400 mg/kg groups compared to the control, 100, and 200 mg MLAE/kg. The highest lipase activity was recorded at 400 mg MLAE/kg, followed closely by the 300 mg/kg group. Protease activity also improved with MLAE supplementation. The 300 and 400 mg/kg groups demonstrated higher protease activity than the control. The differences between 100 and 200 mg MLAE levels and the control were less pronounced.

The total bacterial count decreased significantly in fish fed higher MLAE supplementation levels, with the lowest count observed at 400 mg/kg (Table 4). Fish in the 300 mg/kg group also exhibited lower bacterial counts compared to the control. Similarly, the population of *Vibrio* species and *E. coli* showed a marked reduction in fish served enriched diets with 200, 300, and 400 mg MLAE/kg, with the lowest counts recorded at 400 mg/kg. These reductions were significant compared to the 100 mg MLAE/kg and the control groups. The population of acid-fermentative bacteria remained relatively stable across all treatments, showing no significant changes between control and MLAE-supplemented groups.

### 3.4. Liver and Gastrointestinal Tract Histology

**Figure 4:**
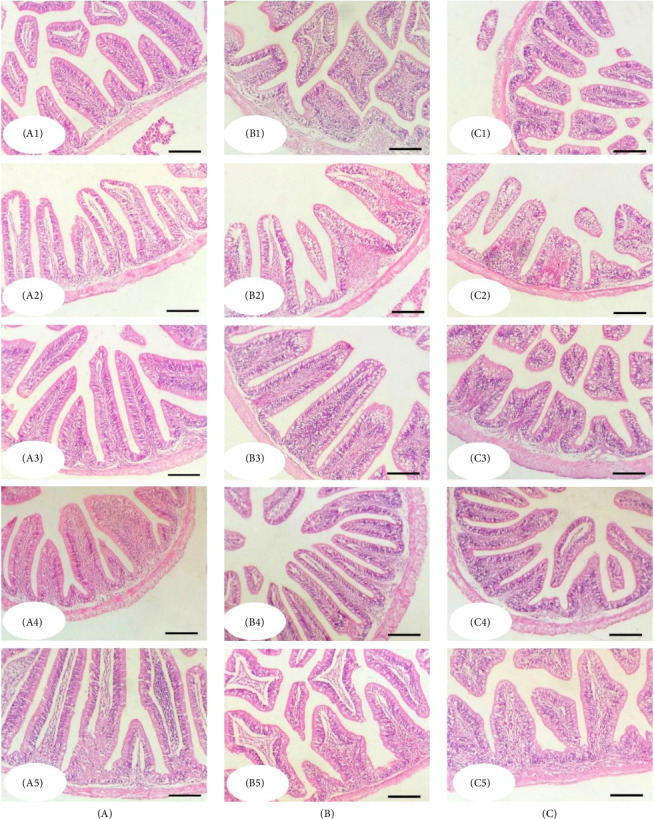
The histomorphology of gray mullet intestine after an 84-day feeding experiment. Stain H&E. Bar = 100 µm. (A1–A5) Anterior intestine; (B1–B5) middle intestine; (C1–C5) posterior intestine. (A1–C1) Control group; (A2–C2) MLAE (100 mg/kg); (A3–C3) MLAE (200 mg/kg); (A4–C4) MLAE (300 mg/kg); (A5–C5) MLAE (400 mg/kg). MLAE, mangrove leaf aqueous extract.

The histological structure of the gray mullet intestine after an 84-day feeding period with diets supplemented with MLAE displayed intact forms of the intestinal wall and intestinal villi in all groups (Figure 4). The intestine was formed of a tunica mucosa of normally arranged simple columnar cells and goblet cells within the intestinal villi, a propria submucosa, and a tunica muscularis and outer serosa. The histological composition of the intestinal villi exhibited a significant enhancement with increased levels of supplemented MLAE (300 and 400 mg/kg). In addition, immune cell infiltration was observed near the intestinal crypts of the middle segments, particularly at the 300 supplementation level (Figure 4B4).

**Figure 5:**
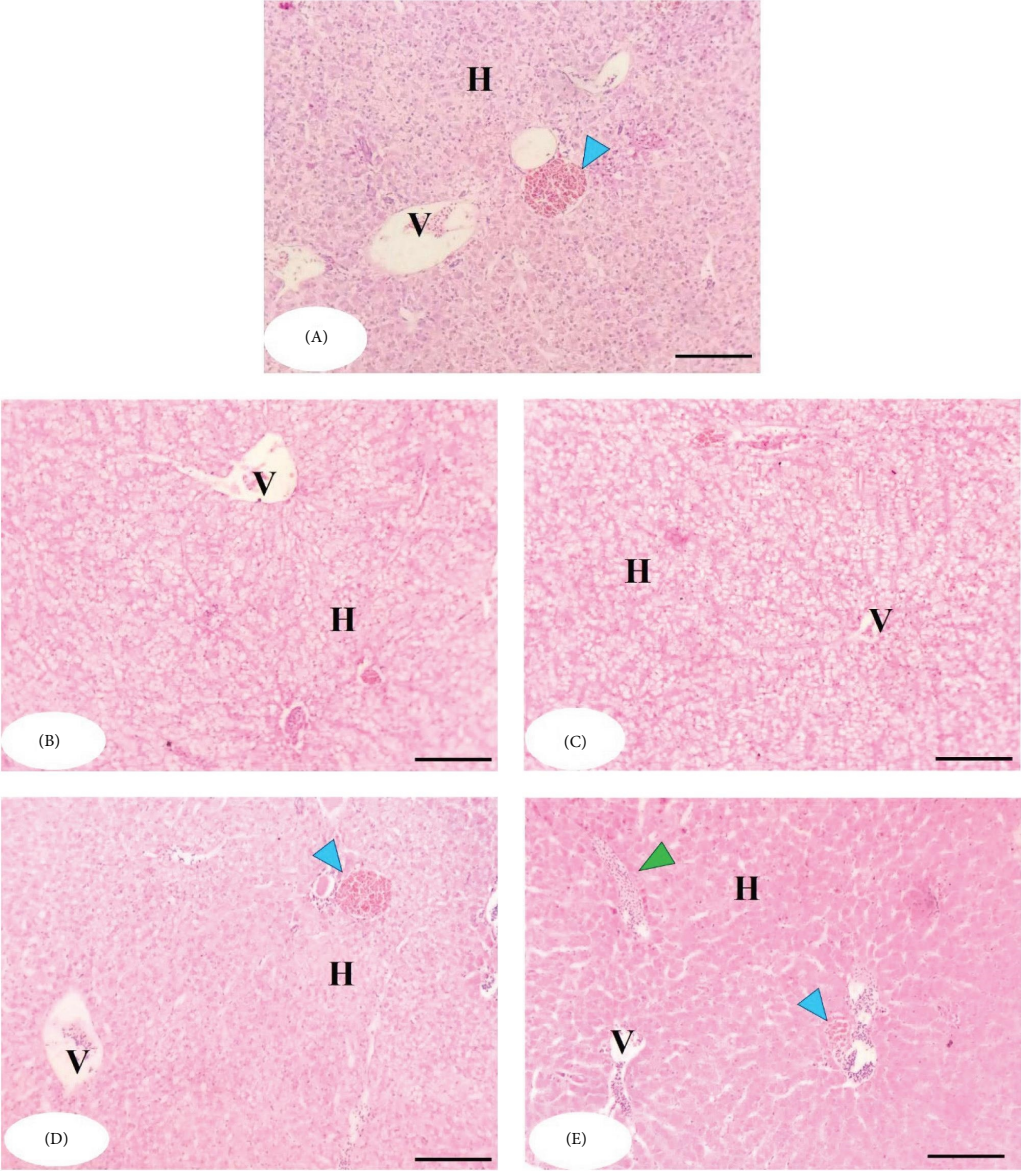
The histomorphology of gray mullet liver after an 84-day feeding experiment. Stain H&E. Bar = 100 µm. (A) Control group; (B) MLAE (100 mg/kg); (C) MLAE (200 mg/kg); (D) MLAE (300 mg/kg); (E) MLAE (400 mg/kg). Blue arrowheads indicate activated melanomacrophages; green arrowheads indicate immune cell infiltration. H, hepatocyte; MLAE, mangrove leaf aqueous extract; V, portal vein.

The histopathological examination of the liver in the MLAE-free group (control) revealed normal hepatic parenchyma, with intact hepatocytes assembled in hepatic cords and separated by normal hepatic sinusoids (Figure 5). The MLAE-supplemented groups triggered an almost enhanced architecture of the hepatic parenchyma (better than the control group) in the form of more well-formed hepatocytes and increased glycogen deposits (Figure 5). The higher levels of MLAE supplementation (300 and 400 mg/Kg) activated melanomacrophages (blue arrowhead) and immune cell infiltration (green arrowhead) (Figure 5D,E).

### 3.5. Serum Chemistry

The serum chemistry of *L. ramada*-fed diets augmented with MLAE at 100, 200, 300, and 400 mg/kg for 84 days are shown in Table 5. The TP levels were augmented in fish-fed diets containing 200, 300, and 400 mg/kg MLAE compared to other groups. The highest TP concentrations were recorded with 300 and 400 mg MLAE/kg levels. Serum AB best levels were observed at 300 and 200 mg MLAE/kg supplementation, respectively. Fish in the 100 mg/kg group exhibited intermediate AB levels, which were substantially higher than the control. Globulin contents were markedly promoted with the 200, 300, and 400 mg MLAE/kg groups compared to the control and 100 mg MLAE/kg groups, with the highest values recorded in the 400 mg/kg group. TC concentrations were reduced (*p* < 0.05) in fish-fed diets containing 200, 300, and 400 mg MLAE/kg compared to the 100 mg/kg and control groups. The lowest cholesterol concentrations were noted in the 300 mg/kg group. TG, ALT, AST, UR, and CR contents remained stable across all treatments.

### 3.6. Immune Indicators

**Figure 6:**
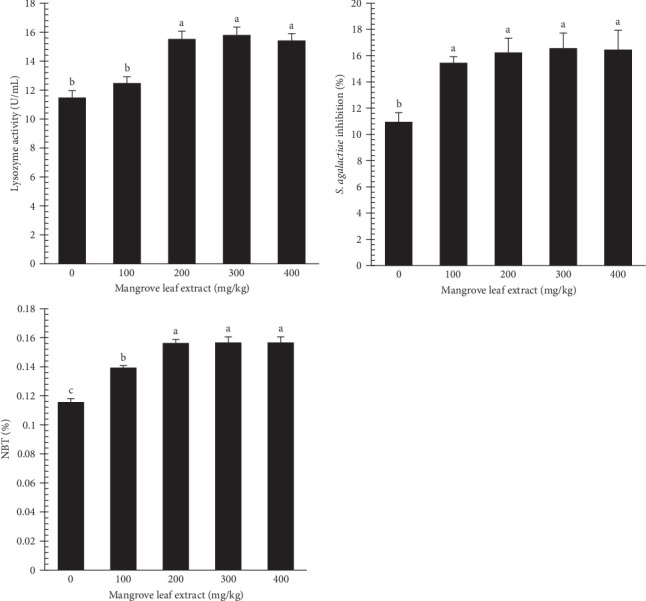
Lysozyme activity, bactericidal %, and NBT% in *Liza ramada* after an 84-day feeding experiment.

Figure 6 illustrates the lysozyme activity, BA, and NBT% in the serum of *L. ramada* after an 84-day trial. Lysozyme activity and NBT% were boosted with higher MLAE content, reaching their highest in the 200–300 mg/kg groups. BA showed significant enhancement at all MLAE supplementation doses compared to the control group.

### 3.7. Antioxidant Responses

**Figure 7:**
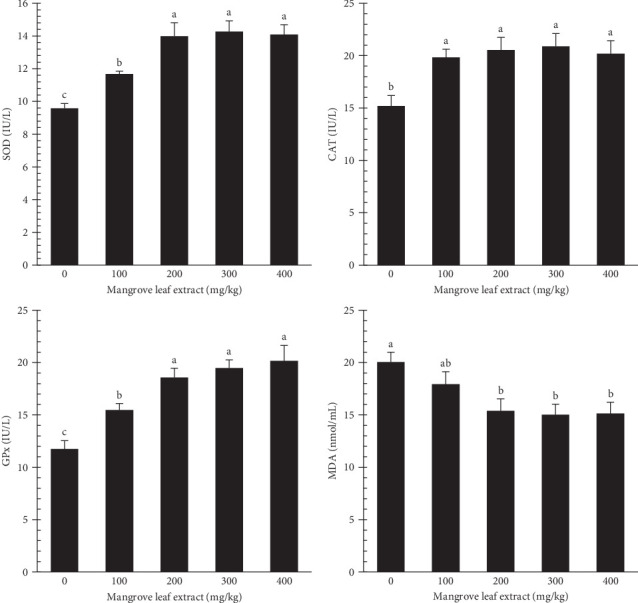
Antioxidant enzymes in *Liza ramada* after an 84-day feeding period. CAT, catalase; GPx, glutathione peroxidase; MDA, malondialdehyde; SOD, superoxide dismutase.

Figure 7 highlights the conditions of antioxidant enzymes (SOD, CAT, and GPx) and the levels of MDA in *L. ramada* after dietary supplementation with MLAE. Antioxidants were promoted in all extract-fed groups, with SOD and GPx showing the highest functions in the 200–400 mg/kg groups. CAT activity was notably increased across all supplemented groups. Conversely, MDA levels decreased progressively with increasing MLAE concentrations, reaching their lowest values in the 200–400 mg/kg groups.

### 3.8. Hypoxia Challenge and Stress Markers

**Figure 8:**
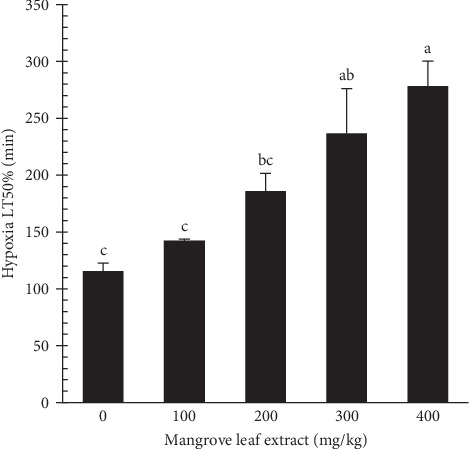
Time to 50% mortality (LT50%, min) of gray mullet-fed mangrove leaf (*Avicennia marina*) aqueous extract incorporated diets for 84-days feeding period. Bars with different letters differ statistically (*p* < 0.05).

The effect of mangrove leaf (*A. marina*) aqueous extract on the time to 50% mortality (LT50%, min) of gray mullet after an 84-day feeding experiment is presented in Figure 8. Fish-fed diets supplemented with MLAE exhibited significantly greater hypoxia tolerance, as indicated by an increased Time to 50% mortality (LT50%), compared to the control group. The control group had the lowest LT50%, while the highest concentration groups (300 and 400 mg MLAE/kg) presented the maximum hypoxia tolerance.

**Figure 9:**
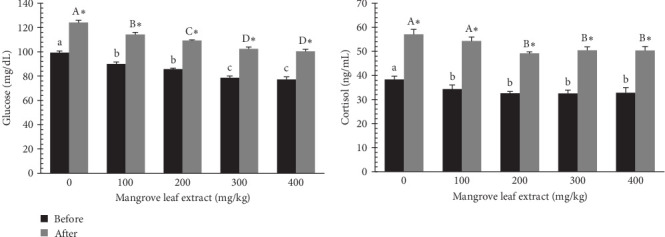
Stress-related markers (glucose and cortisol) of gray mullet-fed mangrove leaf (*Avicennia marina*) aqueous extract incorporated diets for an 84-day feeding period before and after hypoxia stress. Bars with different small letters and capital letters showing significant differences among the groups before or after hypoxia stress (*p* < 0.05). The asterisk (*⁣*^*∗*^) refers to significant differences between the same groups before and after hypoxia stress (*p* < 0.05).

Figure 9 presents the serum glucose and cortisol contents in *L. ramada* before and after exposure to hypoxia, following an 84-day feeding period with diets supplemented with MLAE. Before stress, glucose and cortisol levels decreased with increasing MLAE concentration. After stress, glucose and cortisol levels increased across all groups but remained lower in the extract-fed groups compared to the control.

## 4. Discussion

The incorporation of herbal extracts in aquaculture has garnered significant attention due to the diverse benefits they provide to aquatic species, including mullets. These benefits encompass enhanced immune function, improved antioxidant capacity [11, 35], antibacterial properties [9], as well as increased survival rates and growth performance [10, 36]. The present analysis highlights the rich chemical diversity present in mangrove leaves, showcasing their potential as a source of bioactive components for various applications, such as flavonoids, carotenoids, tannins, phenolic acids, polyphenols, sterols, triterpenes, and triterpenoids. The variety of identified compounds suggests that MLAE could possess diverse pharmacological effects warranting further investigation into specific therapeutic uses. These findings align with previous phytochemical analyses of various mangrove species, including *Rhizophora mangle*, *Laguncularia racemosa* [15, 37], *Avicennia officinalis* [38], *Suaeda maritima* [39], and other mangrove species (*A. marina*, *A. germinans*, *L. racemosa*) [40]. These studies consistently report the presence of flavonoids, carotenoids, polyphenols, and tannins as the predominant phytochemicals, but variations in the concentration and composition of secondary metabolites across different mangrove species may be influenced by ethnobotanical factors [15, 41].

Polyphenolic compounds, a key component of mangrove extracts, are prone to oxidation, forming quinones oxidase, a compound with demonstrated pathogen-killing properties. Quinone oxidase also plays a role in melanin synthesis [42]. The phytochemical profile observed in this study corroborates findings from previous studies, which also reported significant quantities of flavonoids and tannins in mangrove leaf extracts [37, 43]. These compounds are well-documented for their antioxidant and anti-inflammatory properties [44, 45], and their protective effects have been attributed to membrane stabilization. This occurs through charge-binding interactions between the plant extract components and erythrocyte membranes, thereby preventing lysis-induced damage [46].

In aquaculture, the bioactive constituents of MLAE have considerable potential to enhance fish health, as evidenced by the present study. Supplementation with MLAE resulted in improved growth, immune response, and antioxidants in *L. ramada*. Importantly, this study is the first to evaluate the dietary inclusion of MLAE in *L. ramada*, whereas earlier research has focused on the impacts of different mangrove-derived products in other animal species [47]. The uniqueness of this study stems from the utilization of an aqueous extract as a dietary additive. The bioactive composites identified through GC–MS analysis are believed to contribute significantly to alleviating oxidative stress, strengthening immune responses, and stimulating growth factors vital for enhancing aquaculture sustainability and productivity [15].

Growth performance is a critical indicator of fish health and physiological condition. Several studies have verified that herbal supplements can boost appetite and enhance digestive processes, resulting in developed feed utilization and increased growth performance [48, 49]. In this work, dietary supplementation with MLAE improved growth and feed efficiency, with the 300 mg/kg group showing the highest final body weight, weight gain percentage, SGR, FI, and the most efficient FCR. These improvements are likely related to developed digestive enzyme capacity and modulation of gut microbiota. Ali and Bashir [50] observed that supplementation with 1 g/kg body weight of mangrove leaves did not affect rat body weight, while a higher dose of 4 g/kg led to a reduction. This suggests that the effects of plant-based extracts are dose-dependent, influencing physiological outcomes accordingly. Similarly, in this study, fish-fed diets supplemented with 300 and 400 mg/kg MLAE showed significant increases in amylase, lipase, and protease capacities, with the maximum enzyme activity noticed at 400 mg/kg. These findings suggest that bioactive compounds in MLAE, including polyphenols, flavonoids, and tannins, play a crucial role in stimulating digestive enzyme production, thereby enhancing nutrient breakdown and absorption, as supported by previous studies on plant-derived extracts [51]. Elevated amylase and lipase activities enhance carbohydrate and lipid digestion, improving energy availability, while increased protease activity promotes efficient protein digestion, essential for growth and muscle development [52, 53].

Histological analysis of the intestine revealed well-preserved structures across all experimental groups, with the 300 and 400 mg/kg MLAE groups exhibiting increased villus height and width. These structural changes likely contributed to enhanced nutrient absorption and growth performance. The intestinal architecture, comprising normal columnar and goblet cells in the mucosa, as well as a well-formed submucosa, muscularis, and serosa, is consistent with findings from studies on fish-fed herbal extracts [54]. Furthermore, immune cell infiltration near the intestinal crypts, particularly in the 300 mg/kg group, suggests an immunomodulatory effect of MLAE, which may further support overall health and growth [55, 56].

The reduction in total bacterial count, particularly *Vibrio* spp. and *E. coli*, in the 300 and 400 mg/kg groups underscores the antimicrobial role of MLAE. Polyphenols in MLAE are known for their ability to disrupt bacterial membranes and metabolic processes [57, 58]. The decrease in pathogenic bacteria in this study aligns with previous research indicating that plant-derived phenolics reduce bacterial loads in aquaculture species, thus improving gut health and growth performance [59, 60]. Importantly, populations of acid-fermentative bacteria remained constant across all treatments, suggesting that MLAE supplement does not negatively affect the beneficial gut microbiota, which are crucial for fermentation and overall gut homeostasis [61]. Several reports have demonstrated that dietary herbal extracts can influence the gut microbiota of various aquatic species, including *Micropterus salmoides* [62], *Acrossocheilus fasciatus* [63], *Carassius auratus* [64], and *Megalobrama hoffmanni* [65].

The improved growth recorded in the 300 mg/kg group can be credited to better nutrient digestibility and gut health, leading to enhanced feed efficiency. While the 400 mg/kg group exhibited higher enzyme activities and lower bacterial counts, its growth and FCR were slightly lower, suggesting that 300 mg/kg may be the optimal dose for balanced growth and physiological response. These results are consistent with previous studies demonstrating the positive effects of plant extracts on fish growth and gut health [66]. For instance, studies on *Moringa oleifera* leaf extract have shown improvements in digestive enzyme activities and reductions in bacterial loads in *Nile tilapia*, enhancing growth performance [67, 68]. Similarly, a previous study reported that phenolic-rich plant additives improved nutrient digestion and reduced pathogenic bacteria in tilapia, enhancing feed efficiency and health [69]. The antioxidant properties of polyphenols in MLAE likely contribute to reduced oxidative stress, which can impair nutrient metabolism and gut integrity during rapid growth [70].

Dietary bioactive composites are known to influence biochemical keys in fish, which serve as precise indicators of fish health and their responses to environmental stressors [71]. The significant rises in TP, AB, and globulin levels in fish-fed diets containing 200–400 mg/kg MLAE suggest enhanced protein metabolism, liver function, and immune response, likely due to the bioactive substances in MLAE, such as flavonoids, saponins, and phenolic acids [72]. These compounds likely stimulate hepatic protein synthesis and immune globulin production through their antioxidant, anti-inflammatory, and immunomodulatory properties, as seen in studies on *Oreochromis niloticus* and *Labeo rohita* fed plant-based extracts rich in phytochemicals [73, 74]. The antioxidant potential of flavonoids and polyphenols reduces oxidative stress in hepatocytes, facilitating enhanced protein synthesis and immune system activity [75, 76].

This study also revealed that MLAE supplementation improved hepatic architecture, with more well-formed hepatocytes and increased glycogen deposits, suggesting enhanced metabolic activity. Histopathological examination of the liver in the control group showed normal parenchymal structure, while higher MLAE doses (300 and 400 mg/kg) activated melanomacrophages and induced immune cell infiltration, further indicating the immunomodulatory effects of MLAE. Hepatic damage, often associated with histopathological changes such as hepatocyte swelling and cellular vacuolization [77, 78], was notably absent, further highlighting the hepatoprotective effects of MLAE.

Moreover, the reduction in TC levels, particularly in the 300 mg/kg group, supports the hypolipidemic effects of saponins and phenolic acids, which inhibit intestinal cholesterol absorption and regulate biosynthetic pathways by downregulating HMG-CoA reductase activity [12]. Similar cholesterol-lowering effects have been reported in fish and terrestrial animals supplemented with *M. oleifera* extracts, underscoring their role in improving lipid metabolism [79, 80]. The stable ALT and AST levels across all treatments suggest that MLAE supplementation did not induce hepatic damage. ALT and AST are key markers for assessing liver function in fish, with elevated levels indicating hepatic damage or metabolic dysfunction [81]. The stability of these markers, coupled with the enhanced hepatic histology, suggests that MLAE supports liver health and function. The stability of UR and CR levels further confirms the absence of nephrotoxic effects, emphasizing the safety of MLAE supplementation [82]. In line with our results, Al-Harthi et al. [47] reported no major effects on plasma proteins, lipids, or liver enzymes in hens-fed mangrove leaves, though cholesterol decreased slightly, suggesting no harmful impact on metabolic health.

Serum immune markers, which are crucial for assessing the immune health of fish-fed medicinal herbs, showed progressive increases in lysozyme activity and NBT% with higher MLAE supplementation, peaking at 200–300 mg/kg. BA also significantly improved at all MLAE supplementation levels. Increased lysozyme activity, bactericidal capacity, and ACH50 are essential gears of the innate immune system, promoting pathogen cell lysis and enhancing resistance to infections [83]. NBT activity, which interacts with reactive oxygen species (ROS) produced by neutrophils, contributes to non-specific immunity [84]. These findings align with previous studies that indicate enhanced immunity in *L. ramada* when fed medicinal herbs [82, 85, 86]. While the precise mechanism by which mangrove extract acts as an immunomodulator is not fully understood, it is likely that bioactive compounds such as flavonoids, carotenoids, tannins, phenolic acids, polyphenols, sterols, triterpenes, and triterpenoids enhance immune function through the generation of free radicals in neutrophils and increased macrophage phagocytosis [72].

Enhanced immunity in fish is closely associated with improved antioxidative capacity, which protects immune cells from damage caused by ROS by reducing lipid peroxidation and DNA damage [87]. Medicinal herbs, including MLAE, contribute to this antioxidative effect, starting in the intestine and spreading throughout the body via the bloodstream [88]. In this study, antioxidant enzyme activities (SOD, CAT, and GPx) were elevated in MLAE-supplemented groups, particularly at 200–400 mg/kg, while MDA levels progressively declined, reaching their lowest levels in these groups. These outcomes are in line with earlier research on *L. ramada*, where medicinal herbs enhanced antioxidative responses by increasing key antioxidant enzymes [31, 82, 89]. The observed results can be attributed to the antioxidative properties of MLAE, particularly its bioactive compounds, such as saponarin, which act as alpha-glucosidase inhibitors, scavenging ROS and minimizing oxidative stress [42].

Dissolved oxygen levels are critical for aquatic organisms, supporting essential physiological processes such as mitochondrial oxidative phosphorylation [90]. In aquaculture, oxygen levels can fluctuate due to factors such as high stocking densities, algal blooms, organic matter accumulation, and unconsumed feed, which increase the risk of hypoxia [18]. Severe hypoxia disrupts metabolism and physiology, often leading to high mortality [91]. In this study, MLAE supplementation improved hypoxia tolerance, with the highest LT50% observed in the 300 and 400 mg/kg groups. Glucose and cortisol levels decreased with increasing MLAE concentrations before stress and rose after stress across all groups, with MLAE-fed fish maintaining lower levels than the control. These effects are likely attributable to the polyphenols in MLAE, which regulate glycogenesis and reduce cortisol and glucose levels [31, 57]. Hypoxia triggers cortisol-driven glycolysis to generate glucose for energy, but the lower post-stress levels in MLAE-fed fish suggest improved resilience to hypoxia-induced stress [92, 93].

## 5. Conclusion

Dietary supplementation with MLAE significantly enhanced the growth status, feed efficiency, and stress resilience of *L. ramada*. The optimal results were observed at a supplementation level of 300 mg/kg, where growth rates, FCRs, digestive enzyme activity, and immune responses were maximized. Supplementation with MLAE at doses ranging from 300 to 400 mg/kg also advanced antioxidant capacity, reduced oxidative stress markers, and enhanced hypoxia tolerance. Additionally, MLAE supplementation positively influenced blood protein profiles, improved hepatic and intestinal histology, and modulated the gut microbiota by reducing pathogenic bacteria. These findings suggest that MLAE is a promising natural feed additive for improving both aquaculture productivity and fish health, with maximum efficacy observed at 250–300 mg/kg. Future studies should focus on investigating the potential molecular side effects, long-term safety, and the broader applicability of MLAE across different aquaculture species and environmental conditions.

## Figures and Tables

**Table 1:** Ingredients and nutrient profile of the basal diet (% dry weight; *n* = 3).

Ingredients	%
Fish meal (65% CP)	15
Soybean meal (44% CP)	40
Gluten	6
Wheat bran	9
Rice bran	7
Yellow corn	15
Wheat flour	2
Fish oil	3
Soybean oil	1
Mineral premix^a^	0.5
Vitamin premix^a^	0.5
Dicalcium phosphate	1
**Total**	**100**

**Nutrient profile**	

Crude protein (%)	32.23 ± 0.17
Crude lipids (%)	7.19 ± 0.19
Fiber (%)	6.03 ± 0.11
Ash (%)	6.69 ± 0.18
Gross energy (MJ/kg)^b^	18.29 ± 0.28

^a^Vitamin and mineral mixture detailed by Shehata et al. [16].

^b^The gross energy was estimated using the values of 23.6 kJ/g for protein, 39.5 kJ/g for lipid, and 17.2 kJ/g for carbohydrates.

**Table 2:** Mangrove leaf (*A. marina*) aqueous extract constituents.

RT (min)	Compound	Molecular formula	Mass (*M*/*Z*)	Area (%)
5.12	2 Myristynoyl pantetheine	C_25_H_44_N_2_O_5_S	484	2.78
6.1	Tetraacetyl d xylonic nitrile	C_14_H_17_NO_9_	343	2.43
6.33	Akuammilan 17 ol, 10 methoxy	C_20_H_24_N_2_O_2_	324	2.78
6.69	(Meso-tetraphenyl 2,3 secochlorinato 2,3 dialdehyde)nickel (II)	C_44_H_28_N_4_NiO_2_	702	2.43
7.25	2,5 Dibromo 1,4 di n hexadecylbenzene	C_38_H_68_Br_2_	682	2.43
8	Dimethyl 3,3′ [7″ (dimethylcarbamoylmethyl) 8″ [4‴ hydroxybut 1‴ yl) 2″,7″,12″,18″ tetramethyl 7″,8″ dihydro 21 H.23H porphyrin 13″,17″ diyl] dipropionate	C_40_H_51_N_5_O_6_	697	2.43
8.1	{1′,2′ Bis (methoxycarbonyl) 1,1,6,7,11,12 hexamethylbenzo[16,17-d]phthalocyanine}zinc	C_34_H_32_N_4_O_4_Zn	624	1.6
12.53	Methylphosphine D2	CH_3_D_2_P	48	1.6
18.23	(N) (N)′ Dicyclohexyl 1,7 dipyrrolidinylperylene 3,4:9,10 tetracarboxylic acid bisimide	C_44_H_44_N_4_O_4_	692	2.13
18.98	2 Bis (ethoxycarbonyl)methyl 9(2,3,5 tri O (2 methylethylprop 2 yl)dimethylsilyloxy á D ribofuranosyl)purine	C_35_H_64_N_4_O_8_Si_3_	752	1.8
19.21	3,3′ Bis[(5′' tert butylsulfanyl 2″ iodophenyl)ethynyl]tolane	C_38_H_32_I_2_S_2_	806	1.88
22.09	[(1 H) Pyrrole 3 propanoic acid, 2 ethoxycarbonyl 4 ethoxycarbonylmethyl] 5,5′ methylene, bis, diethyl ester	C_33_H_46_N_2_O_12_	662	1.79
22.79	Mo (CO)2[(C4H9 C P)P2]Mo (CO)4[(C4H9 C P)2]	C_21_H_27_Mo_2_O_6_P_5_	726	2.09
25.7	[(Cyclopentadienyl)tris (diethylphosphito P)cobalt O,O′,O″]trichlorozirconium	C_17_H_35_Cl_3_CoO_9_P_3_Zr	730	2.28
26.19	(1 L) (1,2,4/3,5) 1′,2′ anhydro 3,4,5 tri O acetyl 2 (N acetyl N hydroxyamino) 1 hydroxymethyl 3,4,5 cyclopentantriol	C_39_H_47_NO_9_	673	1.97
29.87	1 [2,4,6 Tris (trimethylsiloxy)phenyl] 3 [3,4 di (trimethylsiloxy)phenyl] 2 propen 1 one	C_30_H_52_O_6_Si_5_	648	1.71
30.26	Dichloro (5,10,15,20 tetraphenylporphyrinato)vandium	C_44_H_28_Cl_2_N_4_V	733	1.69
30.4	[(Cyclopentadienyl)tris (diethylphosphito P)cobalt O,O′,O″]trichlorozirconium	C_17_H_35_Cl_3_CoO_9_P_3_Zr	730	2.85
31.15	(2,3 Dihydro 5,10,15,20 tetraphenyl [2 (2)H1]prophyrinato)copper (II)	C_44_H_29_DCuN_4_	677	1.66
32.13	1,3,4,6 Tetra 2 thienylthieno[3,4-c]thiophene	C_22_H_12_S_6_	468	1.64
32.47	ë Chloro 2,4 bis (2 chloroethyl) 6,7 bis[2 (methoxycarbonyl)ethyl] 1,3,5 trimethylporphyrin	C_35_H_37_Cl_3_N_4_O_4_	682	1.81
35.43	YGRKKRRQRRRGP VKRRLDL/5	N/A	N/A	2.27
37.71	Isopropyl ester of 5 endo Methyl 3 oxo tricyclo[5.2.1.0(1,5)]dec 8 en 4 carboxylic acid	C_15_H_20_O_3_	248	1.58
41.27	2,4 Bis (á chloroethyl) 6,7 bis[á methoxycarbonylethyl] 1,3,5 trimethylporphyrin	C_35_H_38_Cl_2_N_4_O_4_	648	1.62
43.33	3,3′,5′ Tri iodo 4′ isopropoxy 6 methoxy 2′ methyl 2 nitrobiphenyl	C_17_H_16_I_3_NO_4_	679	1.65
43.58	Dimethyl anti 14,anti 19 dibromoundecacyclo[9.9.0.0(1,5).0(2,18).0(3,7).0(6,10).0(8,12).0(11,15).0(13,17).0(16,20)]icosane syn 4,syn 9 dicarboxylate	C_24_H_22_Br_2_O_4_	532	2.07
44.33	(2 Nitro 5,10,15,20 tetraphenyl [2 (2)H1]prophyrinato)nickel (II)	C_44_H_26_DN_5_NiO_2_	715	1.73
44.46	5H Cyclopropa (3,4)benz (1,2-e)azulen 5 one, 1,1a à,1b á,4,4a,7a à,7b,8,9,9a decahydro 7b à,9 á,9a à trihydroxy 3 hydroxymethyl 1,1,6,8 à tetramethyl 4a methoxy, 9,9a didecanoate	C_41_H_66_O_8_	686	2.22
45.37	N (4 Dimethylaminophenyl) 3,7,4,8 tetraphenyl 2,6 Imino 2H,6H 1,5 dithiocine	C_32_H_28_Cl_2_O_4_	546	2.19
45.83	4,4′,4″,4‴ Tetrabromotetraphenylmethane	C_25_H_16_Br_4_	632	1.73
46.7	1,2,3,4 Tetrakis (dibromomethyl)benzene	C_10_H_6_Br_8_	758	1.94
47.11	3,3′ Bis[(5″ tert butylsulfanyl 2″ iodophenyl)ethynyl]tolane	C_38_H_32_I_2_S_2_	806	2.41
47.27	(2,3 Dihydro 2 nitro 5,10,15,20 tetraphenyl [3 (2)H1]prophyrinato)copper (II)	C_44_H_28_DCuN_5_O_2_	722	1.8
48.15	5 Silaspiro [4.4]nona 1,3,6,8 tetraene, 3,8 bis (diethylboryl) 2,7 diethyl 1,4,6,9 tetraphenyl	C_44_H_50_B_2_Si	628	1.83
48.42	1,4 Di (5 {[5 (N,N dimethylamino) 1 naphthyl]ethynyl} 1 naphthyl)buta 1,4 diyne	C_52_H_36_N_2_	688	2.45
48.62	(5,10,15,20 tetraphenyl [2 (2)H1]prophyrinato)zinx (II)	C_44_H_27_DN_4_Ni	670	2.23
49.17	Tetra O methyl 3O acetylrobinetinidol (4à,2′)tetra O methyl 3O acetylrobinetinidol 4à acetate	C_44_H_48_O_16_	832	2.22
49.27	YGRKKRRQRRRGP VKRRLFG/5	N/A	N/A	2.9
49.66	(5,10,15,20 tetraphenyl [2 (2)H1]prophyrinato)zinc (II)	C_44_H_28_N_4_Zn	676	2.26
50	2 (2 Carboxyvinyl) 5,10,15,20 tetraphenylporphyrin	C_47_H_32_N_4_O_2_	684	2.31
50.08	(5,10,15,20 tetraphenyl [2 (2)H1]prophyrinato)zinc (II)	C_44_H_28_N_4_Zn	676	1.61
50.3	2,9 Bis (5 tert butyl 2 methoxy 3 pyridylphenyl) 1,10 phenanthroline	C_44_H_42_N_4_O_2_	658	2.02
50.81	Copper tetraphenylporphyrin	C_44_H_28_CuN_4_	675	2.25
51	Ethyl iso allocholate	C_26_H_44_O_5_	436	1.64
51.58	Flavone 4′ OH,5 OH,7 DI O glucoside penitrem A 3	C_27_H_30_O_15_	594	2.25
51.91	Bis (3,6,9,12 tetraoxapentaethylene)crowno N,N,N′,N′ tetramethyl p phanediamine	C_36_H_60_N_4_O_8_	676	2.05
52.07	(2,2 Dibenzyloxy 3 nitro 5,10,15,20 tetraphenyl 2,3 dihydroporphyrinato)copper (II)	C_58_H_40_CuN_5_O_2_	901	1.67
52.16	ë Chloro 2,4 bis (2 chloroethyl) 6,7 bis[2 (methoxycarbonyl)ethyl] 1,3,5 trimethylporphyrin	C_35_H_37_Cl_3_N_4_O_4_	682	1.85
53.42	(2 Nitro 5,10,15,20 tetraphenyl [2 (2)H1]prophyrinato)nickel (II)	C_44_H_27_N_5_NiO_2_	715	2.07
53.7	(2,2 Dibenzyloxy 3 nitro 5,10,15,20 tetraphenyl 2,3 dihydroporphyrinato)copper (II)	C_58_H_40_CuN_5_O_2_	901	2.02

**Table 3:** Growth, feed efficiency, survival rate, and biometric indicis of *L. ramada* performance fed test diets for 84 days.

Parameters	Control	Mangrove leaf aqueous extract (mg/kg)			
		100	200	300	400
Initial body weight (g)	34.96 ± 0.38	34.94 ± 0.02	34.85 ± 0.56	34.80 ± 0.20	34.91 ± 0.52
Final body weight (g)	99.19 ± 1.74^c^	102.88 ± 1.58^c^	115.54 ± 0.43^b^	122.50 ± 2.05^a^	113.24 ± 1.96^b^
Weight gain (%)	183.85 ± 6.79^c^	194.43 ± 4.51^c^	231.67 ± 4.26^b^	251.97 ± 4.53^a^	224.40 ± 6.21^b^
Specific growth rate (SGR, %/day)	1.24 ± 0.03^c^	1.29 ± 0.02^c^	1.43 ± 0.02^b^	1.50 ± 0.02^a^	1.40 ± 0.02^b^
Feed intake (FI, g)	106.24 ± 0.77^c^	110.19 ± 1.22^c^	121.96 ± 2.07^b^	131.38 ± 1.15^a^	121.22 ± 2.09^b^
Feed conversion ratio (FCR)	1.66 ± 0.04^a^	1.62 ± 0.02^a,b^	1.51 ± 0.03^b^	1.50 ± 0.02^b^	1.55 ± 0.06^a,b^
Survival rate (SR, %)	96.67 ± 1.92	97.78 ± 2.22	98.89 ± 1.11	97.78 ± 2.22	98.89 ± 1.11
Hepatosomatic index (HSI, %)	2.00 ± 0.04	2.00 ± 0.04	2.08 ± 0.09	2.08 ± 0.09	2.09 ± 0.09
Intestino-somatic index (ISI, %)	3.47 ± 0.02	3.51 ± 0.03	3.47 ± 0.03	3.41 ± 0.06	3.38 ± 0.08
Visceral somatic index (VSI, %)	6.28 ± 0.06	6.30 ± 0.06	6.25 ± 0.07	6.15 ± 0.07	6.14 ± 0.16
Fulton's condition factor (*K* factor)	2.16 ± 0.09	2.04 ± 0.06	2.04 ± 0.06	2.11 ± 0.05	2.05 ± 0.06

*Note:* Values within the same row are mean ± SE.

^a, b, and c^ denotes significance at *p* < 0.05.

**Table 4:** Digestive enzyme activity and intestinal microbiota of *L. ramada* after an 84-day feeding period on test diets.

Enzyme activity (U/mg)	Control	Mangrove leaf aqueous extract (mg/kg)			
		100	200	300	400
Amylase	15.72 ± 1.19^b^	15.84 ± 1.03^b^	16.19 ± 1.22^b^	19.83 ± 0.32^a^	21.19 ± 0.12^a^
Lipase	19.24 ± 0.32^c^	19.24 ± 0.76^c^	23.67 ± 0.77^b^	26.14 ± 0.35^a^	27.07 ± 0.38^a^
Protease	17.96 ± 0.81^b^	19.46 ± 0.19^a,b^	19.57 ± 0.32^a,b^	20.94 ± 0.17^a^	20.97 ± 1.22^a^
Microbiota count (log CFU/g)					
Total bacterial count	41.67 ± 2.03^a^	38.33 ± 1.20^a,b^	36.00 ± 1.73^a,b^	33.00 ± 2.52^b,c^	29.67 ± 1.76^c^
*Vibrio* sp.	51.67 ± 2.19^a^	45.67 ± 0.88^a^	38.33 ± 1.20^b^	35.67 ± 3.28^b^	33.33 ± 2.60^b^
*E. coli*	16.67 ± 1.20^a^	13.33 ± 0.88^a,b^	12.67 ± 0.88^b^	11.00 ± 1.73^b^	10.33 ± 0.88^b^
Acid-fermentative bacteria	14.33 ± 0.88	13.67 ± 1.76	15.33 ± 1.45	13.33 ± 1.20	13.67 ± 0.88

*Note:* Values within the same row are mean ± S.E.

^a, b, and c^ denotes significance at *p* < 0.05.

**Table 5:** Blood chemistry of *L. ramada* after 84 days on test diets.

Items	Control	Mangrove leaf aqueous extract (mg/kg)			
		100	200	300	400
Total protein (g/dL)	3.38 ± 0.07^b^	3.63 ± 0.07^b^	4.31 ± 0.14^a^	4.49 ± 0.09^a^	4.59 ± 0.12^a^
Albumin (g/dL)	1.25 ± 0.05^c^	1.42 ± 0.03^b^	1.63 ± 0.02^a^	1.70 ± 0.06^a^	1.62 ± 0.04^a^
Globulin (g/dL)	2.13 ± 0.06^b^	2.22 ± 0.06^b^	2.68 ± 0.13^a^	2.79 ± 0.06^a^	2.97 ± 0.16^a^
Total cholesterol (mg/dL)	155.36 ± 5.99^a^	147.54 ± 6.58^a^	111.50 ± 4.93^b^	101.50 ± 2.57^b^	103.55 ± 3.04^b^
Triglyceride (mg/dL)	128.33 ± 5.21	130.67 ± 3.84	129.00 ± 4.04	128.00 ± 2.89	127.67 ± 5.93
ALT (IU/L)	5.49 ± 0.36	5.39 ± 0.41	5.50 ± 0.60	5.55 ± 0.39	5.46 ± 0.46
AST (IU/L)	44.00 ± 1.15	43.67 ± 2.91	42.67 ± 1.86	43.67 ± 3.28	44.67 ± 3.84
Urea (mg/dL)	4.99 ± 0.68	5.06 ± 0.47	5.12 ± 0.50	5.00 ± 0.25	4.79 ± 0.35
Creatinine (mg/dL)	0.29 ± 0.04	0.31 ± 0.03	0.29 ± 0.08	0.31 ± 0.06	0.30 ± 0.05

*Note:* Values within the same row are mean ± S.E.

Abbreviations: ALT, alanine aminotransferase; AST, aspartate aminotransferase.

^a, b, and c^ denotes significance at *p* < 0.05.

## Data Availability

The data set is available from the corresponding authors upon reasonable request.
